# Periostin identified as a potential biomarker of prostate cancer by iTRAQ-proteomics analysis of prostate biopsy

**DOI:** 10.1186/1477-5956-9-22

**Published:** 2011-04-19

**Authors:** Chuanyu Sun, Chao Song, Zhicheng Ma, Ke Xu, Yang Zhang, Hong Jin, Shijun Tong, Weihong Ding, Guowei Xia, Qiang Ding

**Affiliations:** 1 Department of Urology, Huashan Hospital, FudanUniversity, Shanghai, 200040, China; 2 Institute of Psychology, Zhejiang Sci-Tech University, Hangzhou, 310018, China; 3 Institutes of Biomedical Sciences, FudanUniversity, Shanghai, 200032, China

**Keywords:** iTRAQ, Periostin, Proteomics, Prostate cancer

## Abstract

**Background:**

Proteomics may help us better understand the changes of multiple proteins involved in oncogenesis and progression of prostate cancer(PCa) and identify more diagnostic and prognostic biomarkers. The aim of this study was to screen biomarkers of PCa by the proteomics analysis using isobaric tags for relative and absolute quantification(iTRAQ).

**Methods:**

The patients undergoing prostate biopsies were classified into 3 groups according to pathological results: benign prostate hyperplasia (BPH, n = 20), PCa(n = 20) and BPH with local prostatic intraepithelial neoplasm(PIN, n = 10). Then, all the specimens from these patients were analyzed by iTRAQ and two-dimensional liquid chromatography-tandem mass spectrometry (2DLC-MS/MS). The Gene Ontology(GO) function and the transcription regulation networks of the differentially expressed were analyzed by MetaCore software. Western blotting and Immunohistochemical staining were used to analyze the interesting proteins.

**Result:**

A total of 760 proteins were identified from 13787 distinct peptides, including two common proteins that enjoy clinical application: prostate specific antigen (PSA) and prostatic acid phosphatase(PAP). Proteins that expressed differentially between PCa and BPH group were further analyzed. Compared with BPH, 20 proteins were significantly differentially up-regulated (>1.5-fold) while 26 were significantly down-regulated in PCa(<0.66-fold). In term of GO database, the differentially expressed proteins were divided into 3 categories: cellular component(CC), molecular function (MF) and biological process(BP). The top 5 transcription regulation networks of the differentially expressed proteins were initiated through activation of SP1, p53, YY1, androgen receptor(AR) and c-Myc The overexpression of periostin in PCa was verified by western blotting and immunohistochemical staining.

**Conclusion:**

Our study indicates that the iTRAQ technology is a new strategy for global proteomics analysis of the tissues of PCa. A significant up-regulation of periostin in PCa compared to BPH may provide clues for not only a promising biomarker for the prognosis of PCa but also a potential target for therapeutical intervention.

## Background

Prostate cancer(PCa) is the most common cancer and the second leading cause of cancer-related deaths among men in the USA and Europe [1]. It has been estimated that in the USA alone over 192,000 men will be diagnosed with PCa in 2009, causing over 27, 000 deaths [2]. For reasons largely unknown, the incidence of PCa, even when corrected by new widespread testing of serum prostate specific antigen (PSA) has increased in the last two decades [3]. In China, the mortality of prostate cancer also increases every year. A raised PSA level and/or an enlarged or irregular gland on digital rectal examination are used to decide whether transrectal ultrasound -guided biopsy is needed to diagnose PCa [4].

But, PSA can be secreted from benign as well as malignant cells of the prostate so the PSA level is not PCa-specific as it may also be elevated in benign prostate hyperplasia(BPH) and prostatitis [5]. Clinically, 4.0 ng/ml of PSA level is considered as the cutoff value. But, approximately 15% of men with PSA level below 4 ng/ml suffer prostate cancer too. Additionally, it is difficult to distinguish PCa from BPH when PSA level is in the range of 4 to 10 ng/ml known as "diagnostic gray zone". For men with PSA level of 4 to 10 ng/ml, there is about a 25% chance of having PCa [6]. So, PSA presents suboptimum sensitivity and specificity as an early stage marker [7]. Furthermore, Men with PSA level above 4 ng/ml with the diagnosis of BPH confirmed by prostate biopsies still require annual serum test of PSA and repeated biopsies to exclude PCa. It is necessary to develop better means for early diagnosis of PCa [8]. In addition, we are rendered with few effective means to screen patients with potential disease progression who require early intervention from clinically localized PCa [8]. Therefore, the molecular mechanisms of the oncogenesis and progression of PCa needs to be understood in an effort to find new promising biomarkers to make early diagnosis and intervention.

The molecular mechanisms of the development and progression of PCa are complicated and likely to involve multiple factors [9]. In the "seed and soil" hypothesis, it has been pointed out that the interaction between tumor cells and microenvironment plays an important role in oncogenesis and cancer progression [10]. Establishment, growth, and invasion of PCa are supported by this interaction or so called crosstalk in recent studies [11]. Additionally, prostatic intraepithelial neoplasm (PIN) characterized by cellular proliferation within pre-existing ducts, ductules and glands, with cytological changes similar to cancer including nucleus and nucleolar enlargement will be identified in up to 16% of men who have undergone prostate biopsies [12]. More and more studies indicate that high-grade PIN is a premalignant prostate lesion [13]. PCa develops in a series of morphologic and genetic steps that begins with the transformation of normal tissues into hyperplastic lesions, and later into high-grade PIN, invasion and metastasis [14].

Proteomics may help us better understand the changes of multiple proteins involved in oncogenesis and cancer progression and identify more diagnostic and prognostic biomarkers [15]. Quantitative proteomics is an important branch of proteomics which is applied to quantify and identify all the proteins expressed by a whole genome or in a complex mixture. Isobaric tags for relative and absolute quantification(iTRAQ) was developed by Applied Biosystems Incorporation in 2004. It labels global peptide, preserves post-translational modification information and makes quantitative proteomics analysis of 4 samples simultaneously under the same experimental conditions, compared with other approaches such as 2-DE(two-dimensional gel electrophoresis), ICAT (isotop-ecoded affinity tags) and SILAC (stable isotope labeling by amino acids in cell culture) [16,17]. This unique approach labels samples with four independent isobaric tags of the same mass that, upon fragmentation in MS/MS, give rise to four unique reporter ions (m/z from 114 to 117) that provide quantitative information upon integration of the peak areas to quantify the four different samples, respectively [17].

In this study, we analyzed the samples of prostate biopsies from the patients with PCa, BPH, BPH with local PIN by iTRAQ combined with 2DLC-MS/MS (two-dimensional liquid chromatography-tandem mass spectrometry) to find the biomarkers which may contribute to the early diagnosis and prognosis of PCa. 46 proteins were found to demonstrate consistent differential expression between the BPH and PCa samples, among which periostin has been studied in more detail and revealed to be a promising marker of PCa.

## Methods

### Reagents and apparatus

The iTRAQ™ Reagents Kit was from Applied Biosystems (USA). The trypsilin, acetonitrile, isopropanol, acetone, formic acid, glycerol, sodium citrate buffer were from Sigma-Aldrich(USA). KCl, KH2PO4, NaCl, Tris, EDTA, Triton X-100, SDS were obtained from Sinopharm Chemical Reagent Co, Ltd (Shanghai, China). All buffers were prepared with the ultrapure water generated from the Milli-Q system (Millipore, USA). The Sep-Pak Vac C18 cartridges was from Millipore Corporation(USA). 20AD HPLC system (Shimadzu, Japan). The Polysulfoethyl column (2.1 × 100 mm, 5 μm, 300Å; The Nest Group, Southborough, MA), the Zorbax 300SB-C18 reversed-phase column (0.1 × 150 mm, 5 μm, 300 Å; Microm, Auburn, CA) and QSTAR XL System (Applied Biosystem, USA) were used for 2D LC-MS/MS. A rabbit polyclonal antibody of periostin was purchased from Abcam(UK).

### Clinical samples

A total of 11 biopsies have been collected from each of the patients with elevated PSA level or irregular glands by the department of Urology, Huashan Hospital of Fudan University. From these, 10 biopsies were used for standard pathological evaluation and one randomly chosen biopsy from each patient was snap frozen with Tissue-Tek OCT compound in liquid nitrogen, then stored at -80°C until use. The histopathological characteristic was evaluated after standard preparation of 5 μm thick, formalin fixed paraffin embedded sections, stained with hematoxylin and eosin(HE). After pathological evaluation, the patients were categorized in three groups: BPH Group comprised of 20 patients who were diagnosed as BPH with a mean age 67.5 ± 8.8years, mean serum PSA 12.2 ± 6.5 ng/ml. PCa Group included 20 patients who had a diagnosis of PCa with a mean age 70.5 ± 10.6 years, mean serum PSA 26.2 ± 14.0 ng/ml. BPH with local PIN Group had 10 patients diagnosed as BPH with local PIN with a mean age 69.6 ± 10.3years, mean serum PSA 10.3 ± 3.7 ng/mL (range 3.2-19). The PCa group contained 5 patients with PSA level above 20 ng/ml, so, the mean level of PSA in PCa group is higher than that in the other two groups. The study was approved by the local ethics committee of Huashan Hospital of Fudan University.

### Protein preparation and iTRAQ labeling

The eluant of protein samples from the tissues of three groups were quantitated by the Bradford method [18]. The iTRAQ labeling was performed according to the kit prot- ocol. Briefly, 100 μg proteins of each group were precipitated with cold acetone for 1 hour at -20°C and resuspended in 20 μl dissolution buffer. After protein reduction and alkylation followed by overnight digestion with trysilin, the peptides were labeled with the iTRAQ regents for 1 hour at room temperature. The iTRAQ regents 114,116 and 117 were used to label the peptides from BPH, PCa and BPH with local BPH respectively. Then the samples were mixed, desalted with Sep-Pak Vac C18 cartridges and dried in a vacuum centrifuge.

### 2D LC-MS/MS Analysis

The iTRAQ labeling mixed peptides were fractionated by strong cation exchange chromatography (SCX) on a 20AD HPLC system using a Polysulfoethyl column. The peptide mixture was reconstituted in Buffer A(10 mM KH_2_PO4 in 25% acetonitrile, PH 2.6) and loaded onto the column. The peptides were separated at a flow rate of 200 μl/min for 60 min with a gradient of 0-80% Buffer B (Buffer B was Buffer A containing 350 mM KCl) in Buffer A. The absorbance of 214 nm and 280 nm was monitored and a total of 20 SCX fractions were collected. The fractions were vacuum dried and then resuspended in 50 μl HPLC Buffer A (5% acetonitrile, 0.1% formic acid), loaded across the Zorbax 300 SB-C18 reversed-phase column and analyzed on a QSTAR XL System coupled with a 20AD HPLC system. The flow rate of elution was 0.3 μl/min with gradient 5%-35% HPLC Buffer B (95% acetonitrile, 0.1% formic acid) for 90 min. The survey scans were obtained with m/z ranges of 400-1800 for MS with up to three precursors selected from m/z 100-2000 for MS/MS.

### Data analysis

The MS/MS data were searched against the International Swissprot (090210, Human) using the Protein Pilot software (version 3.0, revision 114732, Applied Biosystem, USA) for peptide identification and quantification. The parameters were set as follows:trypsilin as enzyme, methylmethanethiosulphonate of cysteines residues as fixed modification. The Paragon Algorithm (Applied Biosystem, USA) followed by the ProGroup Algorithm (Applied Biosystem, USA) were applied to remove redundant hits to determine the target proteins. Other parameters such as parent ion accuracy, fragment ion mass accuracy, tryptic cleavage specificity, and allowance for number of missed cleavages were provided and processed by ProteinPilot software. Unused ProtScore >1.3 (95%) as threshold with at least more than one peptide above the 95% confidence was considered as benchmark for protein identification. The relative expression of proteins was based on the ratio of the reporter ions of the peptides(116:114 or 116:117). We adopted the fold change of the differentially expressed proteins in the study by Glen et al and the fold change cutoff ratio < 0.66 or >1.50 was selected to designate proteins of differential expression (P < 0.05)[19]. The cellular component (CC), molecular function (MF) and biological process(BP) of the selected proteins were annotated by Gene Ontology (GO) database. The transcription regulation networks of the differentially expressed proteins were generated by MetaCore software using a transcription regulation algorithm. The networks were ranked by a P-value and interpreted in terms of GO.

### Western blotting

To verify periostin expression changes, 5 fresh tissue-samples of BPH and 5 fresh tissue-samples of PCa were analyzed by Western blotting. The tissue samples were lysed in the protein extraction buffer (150 mM NaCl, 10 mM Tris(pH 7.2), 5 mM EDTA, 0.1% Triton X-100, 5% glycerol, and 2% SDS) after tripsis in liquid nitrogen and then incubated at 4°C for 30 min. After centrifugation at 12,000 rpm for 30 min, the protein concentration in tissue homogenate was determined using Bradford assay. Proteins were denatured in sample buffer containing 2-mercaptoethanol and bromophenol blue for 10 min at 95°C. Equal amount of proteins (50 ug) was fractionated using 8 or 12% SDS-PAGE and transferred to PVDF membranes. After blocking with 5% non-fat milk, the membranes were incubated overnight at 4°C with the primary antibody. Then, the membranes washed with PBS three times were incubated in secondary antibody at room temperature. The intensity of target protein was detected using the enhanced chemiluminescence detection system.

### Immunohistochemical staining

Immunohistochemical staining was performed to evaluate the expression of periost-in in 20 paraffin-specimens of BPH and 20 paraffin-specimens of PCa. Each slide was deparaffinized and rehydrated according to standard protocol, and treated with 10 mM sodium citrate buffer in a microwave pressure cooker at 120°C for 15 min. Sections were then immersed in 3% hydrogen peroxide and nonspecified binding was blocked in 5% normal goat serum. A polyclonal anti-periostin was diluted 1:100. Immunohisto-chemical staining was conducted following the avidin-biotin peroxidase complex met-hod with diaminobenzidine as a chromogen. Slides were counterstained with haemat-oxylin, dehydrated and mounted. Brown cytoplasmic staining of stromal or epithelial cells was considered positive. Cytoplasmic and stromal reactivity were analyzed sep-arately. Chi square test was applied to assess the statistical significance of periostin expression in two groups. P-value < 0.05 was considered significant.

## Results

**Figure 1:**
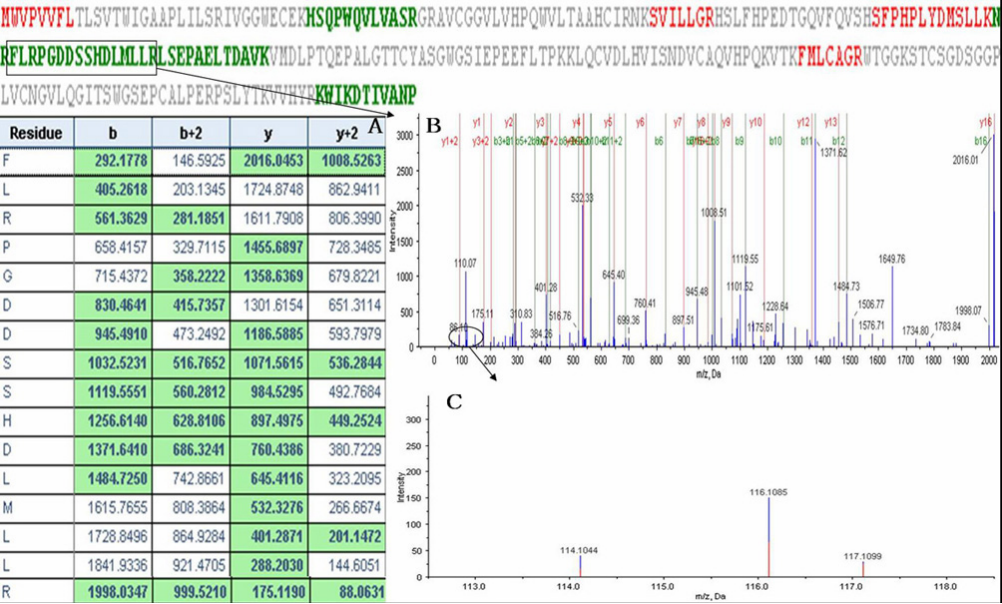
**A representative MS/MS spectrum of PSA**. The relative ratios of PSA between 116(PCa) and 114(BPH) was 1.31. PSA was identified with 11 peptides abo-ve the 95% confidence. This Figure displays the MS/MS spectrum of one peptide from PSA. The peptide sequence: FLRPGDDSSHDLNLLR is shown(The peptides above the 95% confidence are colored green and the peptides in the other colors have lower confidence). BPH samples were labeled with 114 tags, PCa samples were labeled with the 116 tags, and BPH with local PIN samples were labeled with 117 tags. The peptide fragments including b-ion and y-ion series are shown in A and B. The quantitation information of the peptide is shown in C.

**Figure 2:**
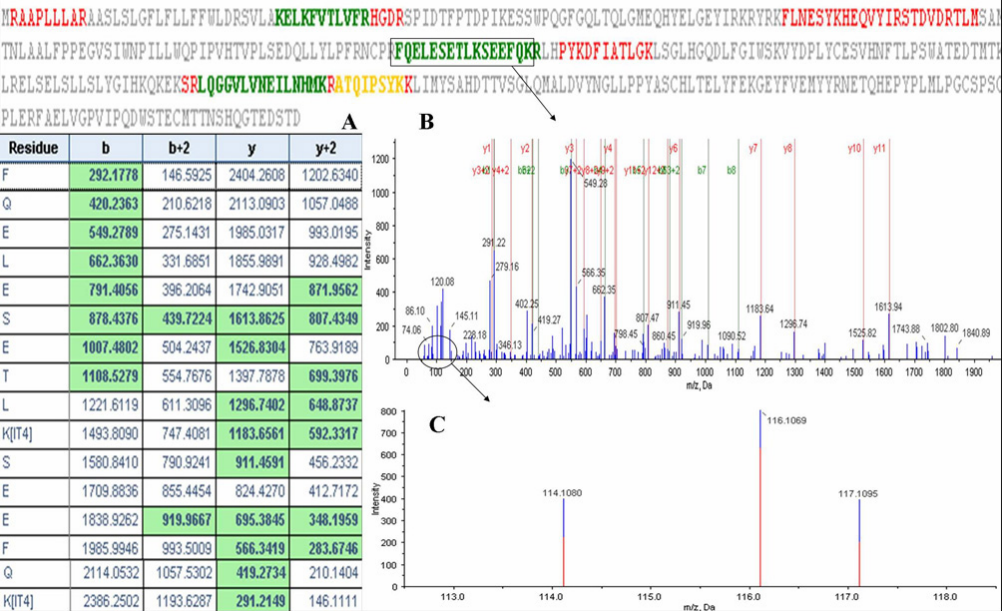
**A representative MS/MS spectrum of PAP**. The relative ratios of PAP between 116(PCa) and 114(BPH) was 1.26. PAP was identified with 4 peptides above the 95% confidence. This Figure displays the MS/MS spectrum of one peptide from PAP. The peptide sequence: FQELESETLKSEEFQK is shown(The peptides above the 95% confidence are colored green and the peptides in the other colors have lower confidence). BPH samples were labeled with 114 tags, PCa samples were labeled with the 116 tags, and BPH with local PIN samples were labeled with 117 tags. The peptide fragments including b-ion and y-ion seriesn are shown in A and B. The quantitation information of the peptide is shown in C.

Based on the condition of protein identification:Unused ProtScore >1.3(95%) as threshold with at least more than one peptide above the 95% confidence, a total of 760 proteins were identified from 13787 distinct peptides. 62% proteins were identified with at least two peptides and 42% proteins were identified with three or more peptides. Actin, gamma-enteric smooth muscle (P63267, Human) was identified with the most peptides above the 95% confidence. There are 191 qualified peptides covering 72.6% of the Actin protein sequence. Among the 760 proteins, PSA (P07288, Human) and Prostatic acid phosphatase(PAP, P15309, Human) were common proteins enjoying clinical application, with the former identified with 11 peptides and the latter 4 peptides above the 95% confidence. Figure 1 and 2 show identification and relative quantitation of peptides from PSA and PAP, respectively.

**Figure 3:**
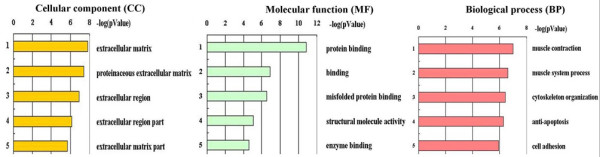
**GO annotation of the differentially expressed proteins**. The differentially expressed proteins were divided into 3 categories: cellular component (CC), molecu-lar function (MF) and biological process(BP). Left: Each enumerated annotation is assi-gned by the enrichment score represented as P value. Right: The top 5 components for CC, MF, BP of the differentially expressed proteins according to GO database are shown.

**Figure 4:**
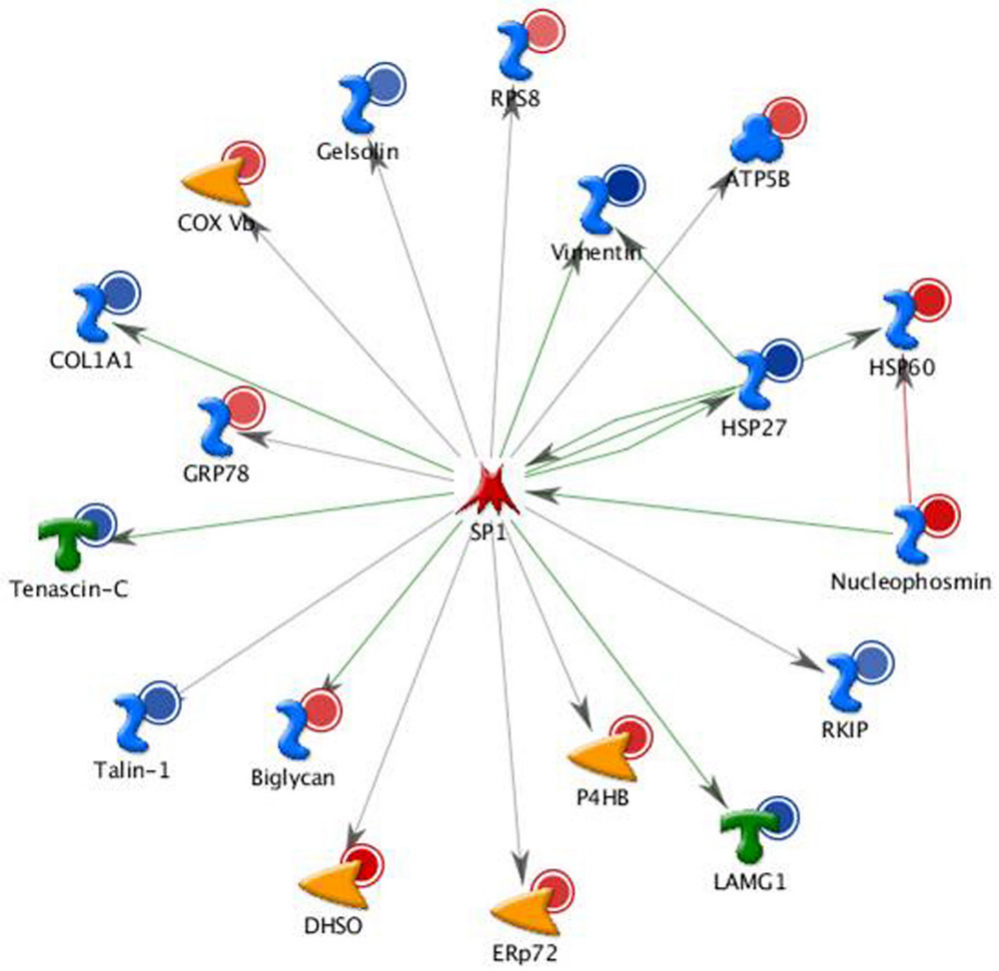
**The transcription regulation network of SP1**. The top 1 transcription regu-lation network initiated through activation of SP1 was shown. SP1 was involved in the regulation of 18 differentially expressed proteins. The up-regulated proteins were indic-ated in red circles and blue circles were used to indicate the down-regulated proteins.

In term of GO database, the differentially expressed proteins were divided into 3 categories: CC, MF and BP. The top 5 components for CC were extracellular matrix, proteinaceous extracellular matrix, extracellular region, extracellular region part and extracellular matrix part. The top 5 components for MF were protein binding, binding, misfolded protein binding, structural molecule activity and enzyme binding. The top 5 components for BP were muscle contraction, muscle system process, cytoskeleton organization, anti-apoptosis and cell adhesion (Figure 3). The transcription regulation networks were ranked in term of the enrichment of the differentially expressed proteins(P-value), rendering the top 5 networks to be the regulation initiated through activation of SP1, p53, YY1, androgen receptor(AR) and c-Myc which were involved in the biological process such as regulation of apoptosis, regulation of cell adhesion, regulation of cellular component organization, response to protein stimulus, etc(The transcription regulation network of SP1 is shown in Figure 4)

Based on the condition of screening differential proteins and compared the BPH samples 114 labeled, 20 proteins were significantly differentially up-regulated and 26 were significantly down-regulated in PCa 116 labeled. Then, from the relative ratio between 116(PCa) and 117(BPH with local PIN), 33 differential proteins can be obtained including 19 up-regulated proteins and 14 down-regulated proteins in PCa. Interestingly, 11 up-regulated proteins and 8 down-regulated proteins were same between the differentially expressed proteins of two sets. In this study, the relative ratio between 116 and 114 was mainly discussed and the differentially expressed proteins were listed in Table 1. Whereas the differentially expressed proteins of PCa compared with BPH with local PIN (116:117) were listed in Table 2. Many of them such as Tumor protein D52, Prohibitin-2, Nucleophosmin, Elongation factor Tu(EF-Tu) have been previously reported as differentially expressed in PCa and closely related to oncogenesis and cancer progression [15,20-22]. Interestingly, the down-regulated proteins in our study included several differentiation markers of smooth muscle(SM) such as Desmin, Vimentin, Actin, gamma-enteric smooth muscle, Laminin subunit gamma-1, Vinculin which were studied by Wong et al [23]. Our results of down-regulated proteins were consistent with the changes of the SM differentiation markers from their study.

**Table 1:** Differentially expressed proteins(PCa:BPH)

Accession	Protein name	Peptides(95%)	116:114
Down-regulation			
sp|P17661	**Desmin**	62	0.03
sp|O95810	**Serum deprivation-response protein 3**	3	0.13
sp|Q9BX66	**Sorbin and SH3 domain-containing protein 1**	18	0.18
sp|P08670	Vimentin	65	0.19
sp|P51911	**Calponin-1**	43	0.20
sp|P04792	Heat shock protein beta-1	15	0.22
sp|P63267	Actin, gamma-enteric smooth muscle	191	0.24
sp|Q969G5	**Protein kinase C delta-binding protein**	6	0.24
sp|P12109	Collagen alpha-1(VI) chain	19	0.25
sp|P11047	Laminin subunit gamma-1	9	0.28
sp|O94875	Sorbin and SH3 domain-containing protein 2	2	0.32
sp|P24821	Tenascin	7	0.35
sp|Q15942	**Zyxin**	4	0.35
sp|Q9Y490	**Talin-1**	21	0.36
sp|P02452	Collagen alpha-1(I) chain	92	0.38
sp|P18206	**Vinculin**	15	0.39
sp|P20774	Mimecan	6	0.40
sp|Q93052	Lipoma-preferred partner	12	0.41
sp|Q09666	Neuroblast differentiation-associated protein AHNAK	62	0.42
sp|P07585	Decorin	12	0.43
sp|P98160	Basement membrane-specific heparan sulfate proteoglycan core protein	17	0.44
sp|P30086	Phosphatidylethanolamine-binding protein 1	7	0.47
sp|P06396	Gelsolin	20	0.49
sp|P12111	Collagen alpha-3(VI) chain	63	0.50
sp|P01834	Ig kappa chain C region	4	0.53
sp|P10909	Clusterin	3	0.54
Up-regulation			
sp|P62241	40S ribosomal protein S8	2	1.53
sp|P31943	**Heterogeneous nuclear ribonucleoprotein H**	3	2.17
sp|P11021	**78 kDa glucose-regulated protein**	27	2.21
sp|P09669	Cytochrome c oxidase polypeptide VIc	2	2.38
sp|P10606	**Cytochrome c oxidase subunit 5B, mitochondrial**	7	2.70
sp|P06576	ATP synthase subunit beta, mitochondrial	13	2.83
sp|P38646	Stress-70 protein, mitochondrial	11	2.91
sp|P21810	**Biglycan**	19	2.96
sp|P49411	**Elongation factor Tu, mitochondrial**	6	3.02
sp|P22314	Ubiquitin-like modifier-activating enzyme 1	5	3.34
sp|P13667	**Protein disulfide-isomerase A4**	5	3.77
sp|Q5SSJ5	Heterochromatin protein 1-binding protein 3	5	4.06
sp|P55327	Tumor protein D52	3	4.25
sp|Q99623	Prohibitin-2	3	4.57
sp|P07237	**Protein disulfide-isomerase**	17	4.70
sp|Q9NZN4	EH domain-containing protein 2	2	5.55
sp|P10809	**60 kDa heat shock protein, mitochondrial**	25	5.97
sp|P06748	**Nucleophosmin**	10	7.94
sp|Q15063	**Periostin**	13	9.12
sp|Q00796	**Sorbitol dehydrogenase**	9	9.82

*The proteins written with bold words were the same differentially expressed proteins between 116:114(PCa:BPH) and 116:117(PCa:BPH with local PIN).

**Table 2:** Differentially expressed proteins(PCa:BPH with local PIN)

Accession	Protein name	Peptides(95%)	116:117
Down-regulation			
sp|P17661	**Desmin**	62	0.03
sp|P24844	Myosin regulatory light polypeptide 9	10	0.14
sp|O95810	**Serum deprivation-response protein 3**	3	0.16
sp|Q9BX66	**Sorbin and SH3 domain-containing protein 1**	18	0.20
sp|P09493	Tropomyosin alpha-1 chain	60	0.21
sp|Q969G5	**Protein kinase C delta-binding protein**	6	0.28
sp|P51911	**Calponin-1**	43	0.29
sp|P18206	**Vinculin**	15	0.30
sp|P15309	Prostatic acid phosphatase	4	0.32
sp|Q15942	**Zyxin**	4	0.36
sp|P02671	Fibrinogen alpha chain	18	0.37
sp|P07305	Histone H1.0	6	0.37
sp|Q9Y490	**Talin-1**	21	0.50
sp|P30101	Protein disulfide-isomerase A3	33	0.58
Up-regulation			
sp|Q09666	Neuroblast differentiation-associated protein AHNAK	62	1.91
sp|P00367	Glutamate dehydrogenase 1, mitochondrial	10	2.19
sp|P13667	**Protein disulfide-isomerase A4**	5	2.19
sp|P10606	**Cytochrome c oxidase subunit 5B, mitochondrial**	7	2.31
sp|P12111	Collagen alpha-3(VI) chain	63	2.33
sp|P31943	**Heterogeneous nuclear ribonucleoprotein H**	3	2.42
sp|P61604	10 kDa heat shock protein, mitochondrial	9	2.44
sp|P49411	**Elongation factor Tu, mitochondrial**	6	2.86
sp|P11021	**78 kDa glucose-regulated protein**	27	2.93
sp|P35579	Myosin-9	29	3.25
sp|P07237	**Protein disulfide-isomerase**	17	3.44
sp|P21810	**Biglycan**	19	3.53
sp|P22392	Nucleoside diphosphate kinase B	5	3.73
sp|P10809	**60 kDa heat shock protein, mitochondrial**	25	5.55
sp|Q15063	**Periostin**	13	5.55
sp|P62906	60S ribosomal protein L10a	6	5.97
sp|P06748	**Nucleophosmin**	10	6.37
sp|Q9BXN1	Asporin	7	7.38
sp|Q00796	**Sorbitol dehydrogenase**	9	9.12

*The proteins written with bold words were the same differentially expressed proteins between 116:114(PCa:BPH) and 116:117(PCa:BPH with local PIN).

**Figure 5:**
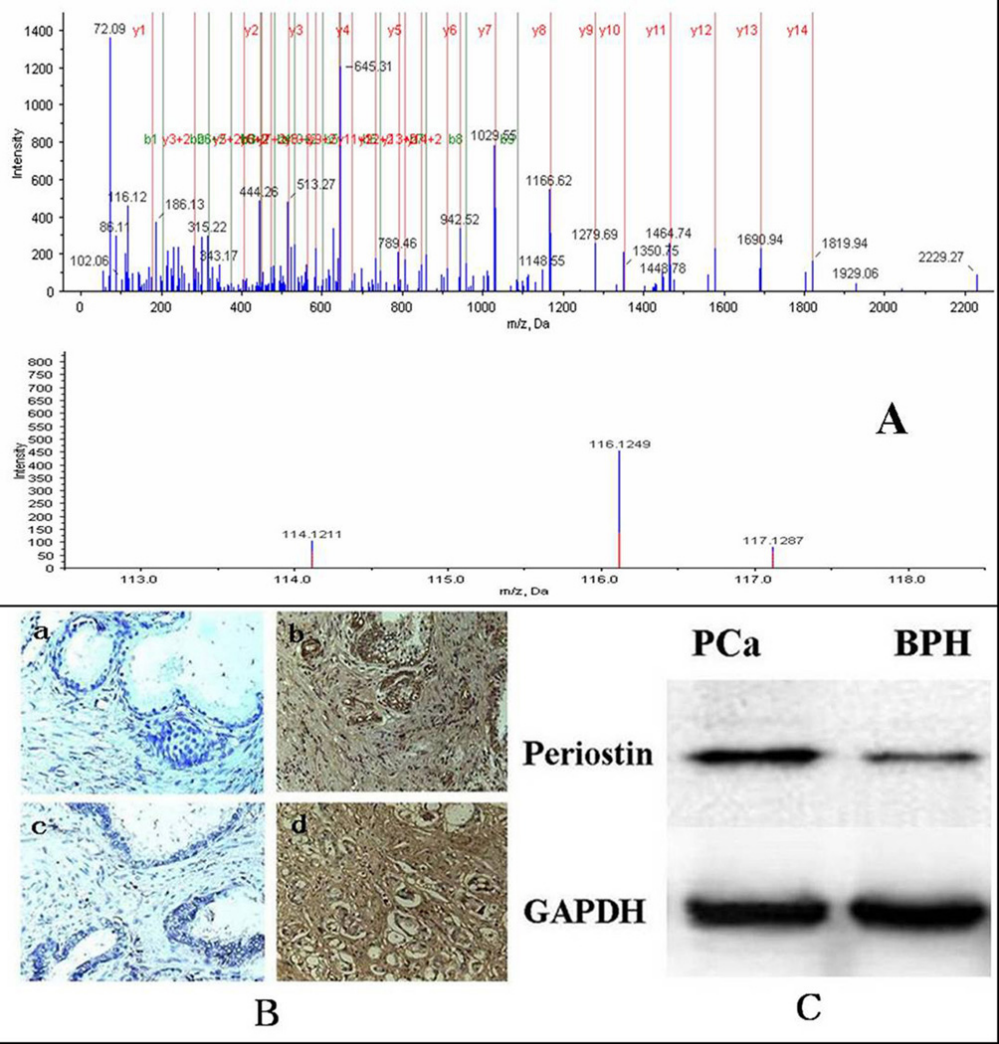
**The quantitation information of one peptide from periostin and the expression of periostin in malignant and benign prostate tissue**. A:The quantitation information of the peptide: GLESNVNVELLNALHSHMNKR from periostin. BPH samples were labeled with114 tags, PCa samples were labeled with the 116 tags, and BPH with local PIN samples were labeled with 117 tags. B: Immunohistochemical staining of periostin in PCa and BPH. Negative epithelial and stromal periostin expression in BPH(a) and PCa(c). Positive epithelial and stromal periostin expression in BPH(b) and PCa(d). C: The results of western blotting revealed a significant increase of periostin amount in PCa compared to BPH(P < 0.05).

Among the identified proteins, periostin was an interesting protein showing up-regulation in PCa and was further studied. Figure 5A shows the identified information of periostin. The expression of periostin was verified by western blotting. The results revealed a significant increase of periostin amount in PCa compared to BPH (Figure 5C). Furthermore, immunohistochemical staining was performed to evaluate periostin expression in the stromal or epithelial cells of prostate (Figure 5B). Benign prostate glands expressed positive stromal periostin in only 5/20 cases and positive epithelial periostin in 8/20 cases. The stroma of PCa was positive in 16/20 cases and the epithelium of PCa was positive in 12/20 cases. Statistical significance was observed for the stromal expression of periostin between PCa and BPH(P < 0.01). However, there was no statistical significance for the epithelial expression of periostin between PCa and BPH (Table 3).

**Table 3:** Epithelial and stromal expression of periostin in PCa and BPH

	Epithelial expression			Stromal expression		
		
	Negative	Positive	P value	Negative	Positive	P value
PCa	8 (40%)	12 (60%)		4 (20%)	16(80%)	
BPH	12(60%)	8 (40%)	0.206	15 (75%)	5 (25%)	P < 0.01

## Discussions

Although PSA has played a great role in diagnosing PCa, the sensitivity and specificity of PSA have been questioned by more and more studies [5-7]. So, many researchers have applied the proteomic technologies to search for new biomarkers, furthermore, a large number of differentially expressed proteins have been identified and some were reported as potential biomarkers for diagnosis and prognosis of PCa [11,15,19-21,24]. However, most of the studies were related to Western. It is well recognized that the incidence of PCa varies widely among ethnic populations and countries [25]. In this study, we focused proteomics analysis on the tissues obtained from prostate biopsies to identify new biomarkers of PCa in Chinese the population.

The development of the iTRAQ shot-gun proteomic approach has offered the option to study differential expression of proteins in perturbed systems. It can provide quantitative information from numerous experimental approaches including affinity pull-downs, time-course analyses, and discovery and confirmation of disease markers [16]. So far, iTRAQ has been used in studying the proteomics of the tissue and the cell line of PCa [15,19]. Garbis et al [15] conducted a study on 20 patients: 10 BPH and 10 PCa patients, and utilized iTRAQ combined with 2DLC-MS/MS to identify 825 proteins. Of the differentially expressed proteins, 30 were shown to be up-regulated and 35 were down-regulated in PCa. In their study, the samples were obtained from postoperative tissues. We agreed with the opinion that proteomics analysis of prostate biopsies would enable biomarker investigations of pathologically characterized clinical samples [20]. So, we chose the samples of prostate biopsy to study. Compared with the study of Garbis, there were five same differentially expressed proteins, of which, periostin and nucleophosmin were up-regulated and the others were down-regulated including collagen α-1(VI), zyxin, and vinculin. Additionally, In our study, the two well known and clinically applied proteins PSA and PAP were identified, a strong proof of the reliability of iTRAQ approach in proteomics analysis of PCa(Figure 1 and 2). The relative ratios of PSA and PAP between 116(PCa) and 114(BPH) were 1.31 and 1.26, respectively. But, based on the condition of screening the differentially expressed proteins, there were no difference of PSA and PAP between PCa(116) and BPH(114), Additionally, PAP was reversely down-regulation in the study of Garbis. We considered that the difference may partly be due to the different experiemental conditions and experiemental errors.

Of the up-regulated proteins, subcellular location of six proteins is mitochondria. They are Cytochrome c oxidase polypeptide VIc(COX6C), Cytochrome c oxidase subunit 5B(COX5B), ATP synthase subunit β(ATP5B), Stress-70 protein(HSPA9), EF-Tu, 60 kDa heat shock protein(HSPD1). Mitochondria have dual functions in carcinogenesis, namely, cancer-associated changes in cellular metabolism: the Warburg effect and the apoptosis-linked mitochondrial permeability transition pore [26] Alterations in mitochondrial structure and function may contribute to development of PCa. Cytochrome c oxidase(COX) composed of 13 individual protein subunits is at the heart of oxidative metabolism. Both COX6C and COX5B are the subunits of COX which are synthesized from nuclear DNA found on a variety of chromosomes [27]. Krieg etal [27] reported that there was an associated increase in the ratio of nuclear encoded COX subunits to mitochondrially encoded COX subunits in the tumor-derived cell lines including PCa cells. ATP synthase is required for cellular energy production. The expression of ATP synthase mRNA in several malignant tumors including PCa is up-regulated compared with the normal tissue [28]. ATP synthase is reduced when a metastatic human prostate cell line is converted to a slow growing cell line [29] and down-regulated upon treatment of prostate cancer cell lines with rapamycin [30]. Heat shock proteins (HSPs) protect cells against stress-associated injury and are overexpressed in several malignant tumors including PCa. So, HSPs have been considered useful as diagnostic or prognostic predictive factors in a variety of tumors. Furthermore, inhibiting the function of HSPs in tumor cells as an attractive strategy in cancer treatment has been reported [31]. EF-Tu belongs to EF-Tu/EF-1A subfamily of the GTP-binding elongation factor family and promotes the GTP-dependent binding of aminoacyl-tRNA to the A-site of ribosomes(http://www.uniprot.org/). A proteomics analysis about gastric carcinoma cell line revealed high expression of EF-Tu [26]. The up-regulation of EF-Tu was also detected in PCa by proteomics analysis [21]. It has been reported that the proliferation, invasion and migration of PCa Cells were inhibitied by down-regulating EF-1A expression [9]. Our results provide clues as to the mechanism of the mitochondrial changes at the protein level in PCa. So, the mitochondrial proteins may serve as potential biomarkers of PCa.

The proteins found to be down-regulated in our study included several differentiation markers of smooth muscle(SM) such as Desmin, Vimentin, Actin, gamma-enteric smooth muscle, Laminin subunit gamma-1, Vinculin. All these SM differentiation marks had been studied by Wong etal [23]. In their study, immunohistochemical methods revealed that the expression of SM markers was markedly subdued and varied in the well-differentiated tumors, whereas SM were totally absent in the moderately to poorly differentiated tumors, except in regions around vasculature. Our results of down-regulated proteins were consistent with the changes of the SM differentiation markers from their study. Interestingly, heterogeneous nuclear ribonucleoprotein-H (hnRNP-H) that plays a suppressive role in SM myogenesis is up-regulated in our study [32]. This may have revealed the mechanism of SM-epithelial interactions in carcinogenesis of PCa.

It is better and easier to understand the location, function and regulation of the differentially expressed proteins by bioinformatics analysis. The top 5 components for each category of GO in Figure 3 indicated that the differentially expressed proteins in our study mainly located in extracellular matrix with the function of binding were involved in muscle related BP, cytoskeleton organization, anti-apoptosis and cell adhesion. Additionally, the top 5 regulation networks indicated that the SP1, p53, YY1, androgen receptor(AR) and c-Myc were 5 important factors regulating the differentially expressed proteins. In other words, the 5 transcription factors may play great roles in the oncogenesis and progression of PCa.

Periostin, also named osteoblast-specific factor 2(OSF-2), which was originally identified in the mouse osteoblastic cell line:MC3T3-E1 as a secreted matricellular protein [33]. The sequence of periostin contain a typical signal sequence, a cysteine-rich domain, a fourfold fasciclin 1-like (FAS-1) domain and a C-terminal domain [33,34]. The FAS-1 domain, an evolutionarily ancient adhesion domain also exists in many proteins such as big-h3, stabling I and II, MBP-70, algal-CAM and periostin-like factor. Therefore, all those proteins containing periostin with the FAS-1 domain belong to the fasciclin family [35]. Additionally, periostin share high homology in human and mouse with 89.2% overall amino acid identity and 90.1% identity in their mature forms [36]. The periostin gene is located on chromosome 3 in mouse, while the human periostin gene is located on chromosome 13q encoding a protein of 835 amino acids with a molecular weight of 90 kDa [37].

Periostin can interact with other extracelluar matrix scaffold proteins, such as fibronectin, tenascin C, collagen type I, collagen type V and heparin. It has been discovered to be a ligand for αvβ3 and αvβ5 integrins inducing integrin-dependent cell adhesion and motility [38]. Being highly expressed in periosteum, perichondrium, periodontal ligaments, the fascia of muscles, articular surfaces of the epiphyseal cartilage and joint ligaments [39,40], periostin is thought to play a potential role in formation and structural maintenance of those tissues [40]. Additionally, it has been reported that periostin expression is correlated with the developing and the diseased heart [41].

Recently, periostin was found to be overexpressed in various types of human cancers including non-small-cell lung cancer, breast cancer, ovarian cancer, colon cancer, head and neck cancer, pancreatic cancer, liver cancer and neuroblastoma [36]. It is thought that periostin enhances tumor growth through multiple pathways such as promoting cell proliferation, evasion of apoptosis, limitless replicative potential, genomic instability and induction of angiogenesis [36]. Additionally, periostin overexpression is always correlated with increased tumor invasion and metastasis [42]. It is indicated that periostin can facilitate tumor invasion and metastasis by the process of epithelial-mesenchymal transition(EMT) which enables epithelial cancer cells to acquire invasive and metastatic potential [36]. So far, Only two studies have reported that the high stromal expression of periostin was clearly and significantly correlated with high grade and high stage of PCa [43,44]. In our study, periostin was screened by proteomics analysis of the tissues of prostate biopsy. Then, the overexpression of periostin in PCa was vertified by western blotting and immunohistochemical staining(Figure 5). Our results of immunohistochemical staining indicated that high expression of periostin was mainly present in the stroma of PCa(Table 3). Based on the result of this study along with the above two studies, periostin may become a promising biomarker for the diagnosis and prognosis of PCa.

## Conclusion

Our study indicates that the iTRAQ technology is a new strategy for global proteomics analysis of the tissues of PCa. A significant up-regulation of periostin in PCa compared to BPH may offer clues of not only a promising biomarker for the prognosis of PCa but also a potential target for therapeutical intervention. However, the role of periostin in the development and invasion of PCa is in need of further in-depth study.

## Abbreviations

PCa: prostate cancer; BPH: benign prostate hyperplasia; PIN: prostatic intraepithelial neoplasm; PSA: prostate specific antigen; PAP: prostatic acid phosphatase; 2DLC-MS/MS: two-dimensional liquid chromatography-tandem mass spectrometry; GO: Gene Ontology; TPD52: Tumor protein D52; SM: smooth muscle.

## Competing interests

The authors declare that they have no competing interests.

## Authors' contributions

CSun and KX performed the experiments. GX and QD designed the experiments and were co-corresponding author. ST, WD and ZM collected the samples. CSong, YZ and HJ analyzed the data. CSun drafted the manuscript. All authors read and approved the final manuscript.
